# Consistency vs. switching triptan treatment and headache-related disability: Results of the American migraine prevalence & prevention study

**DOI:** 10.1186/1129-2377-1-S14-P212

**Published:** 2013-02-21

**Authors:** DC Buse, D Serrano, S Kori, C Cunanan, AN Manack, ML Reed, RB Lipton

**Affiliations:** 1 Albert Einstein College of Medicine, USA; 2 Vedanta Research, USA; 3 MAP Pharmaceuticals, USA; 4 Allergan Inc., USA

## Background

Switching from a triptan to another acute medication may be an indicator of unmet treatment need.

## Objectives

To quantify changes in headache-related disability for migraineurs who switched from a triptan to another acute treatment in a population-based sample.

## Methods

AMPP study surveys were mailed to a sample of 24,000 persons with "severe headache" identified in 2004 and followed annually through 2009. Eligible subjects had ICHD-2 migraine, reported triptan use one year and medication data in the subsequent year (a couplet). We examined 4 patterns: a) consistent triptan use, b) switching to another triptan, c) switching to an opioid/barbiturate, or d) switching to a NSAID. Change in disability was measured with MIDAS change from the second to the first year (negative change scores reflect reduction in disability). Change scores were modeled via ANOVA for couplets by 3 strata of average headache days: low (0-4 days/month), moderate (5-9 days/month), and high frequency episodic/chronic migraine ([HFEM/CM]≥10 days/month). ANOVAs were estimated for each switch pattern relative to the consistent triptan use group. The values of (b) represent change in MIDAS score.

## Results

146 respondents met inclusion criteria and reported a switch pattern of interest. Mean MIDAS change in the consistent triptan use group was -7.4. For those switching to another triptan the mean change was -3.5 (NS difference between groups; b=4.0, p=0.23). For those switching to an opioid or barbiturate the mean change was 3.4 (NS difference compared to consistent group; b=10.9, p=0.079). For those switching to an NSAID, the mean change was 10.3, reflecting significantly higher disability (b=17.7, p=0.002), particularly among those with HFEM/CM (b=31.7, p=0.004).

## Conclusion

Switching from a triptan to another triptan, an opioid or barbiturate was not associated with significant improvement in headache-related disability relative to consistent triptan use. Switching to a NSAID was associated with an increase in disability; especially among those with HFEM/CM.

